# Low-cost MEMS accelerometers for earthquake early warning systems: A dataset collected during seismic events in central Italy

**DOI:** 10.1016/j.dib.2024.110174

**Published:** 2024-02-07

**Authors:** Marco Esposito, Simone Marzorati, Alberto Belli, Chiara Ladina, Lorenzo Palma, Carlo Calamita, Debora Pantaleo, Paola Pierleoni

**Affiliations:** a Department of Information Engineering (DII), Università Politecnica delle Marche, Ancona 60131, Italy; b Istituto Nazionale di Geofisica e Vulcanologia (INGV), Osservatorio Nazionale Terremoti, 60131 Ancona, Italy

**Keywords:** Internet of things, Wireless sensor network, MEMS accelerometers, Structural health monitoring, Earthquake early warning

## Abstract

This article describes a dataset of acceleration signals acquired from a low-cost Wireless Sensor Network (WSN) during seismic events that occurred in Central Italy. The WSN consists of 5 low-cost sensor nodes, each embedding an ADXL355 tri-axial MEMS accelerometer with a fixed sampling frequency of 250 Hz. The data was acquired from February 2023 to the end of June 2023. During this period, several earthquake sequences affected the area where the sensor network was installed. Continuous data was acquired from the WSN and then trimmed around the origin time of seismic events that occurred near the installation site, close to the city of Pollenza (MC), Italy. A total of 67 events were selected, whose data is available at the Istituto Nazionale di Geofisica e Vulcanologia (INGV) Seismology data center. The traces acquired from the WSN were then manually annotated by analysts from INGV. Annotations include picking time for P and S phases, when distinguishable from the background noise, alongside an associated uncertainty level for the manual annotations. The resulting dataset consists of 328 3 × 25,001 arrays, each associated with its metadata. The metadata includes event data (hypocenter position, origin time, magnitude, magnitude type, etc.), trace-related data (mean, median, maximum, and minimum amplitudes, manual picks, and picks uncertainty), and sensor-specific data (sensor name, sensitivity, and orientation). Furthermore, a small dataset consisting of non-seismic traces is included, with the goal of providing records of noise-only traces, relative to both electronic and environmental/anthropic noise sources.

The dataset holds potential for training and developing Machine Learning or signal processing algorithms for seismic data with low signal-to-noise ratios. Additionally, it is valuable for research about earthquakes, structural health monitoring, and MEMS accelerometer performance in civil and seismic engineering applications.

Specifications Table

**Table:** 

Subject	Computers in Earth Sciences
Specific subject area	Seismology
Type of data	Digital time-series
How the data were acquired	Acquisition campaign using a low-cost wireless sensor network composed by 5 ADXL355 tri-axial MEMS accelerometers.
Data format	Analyzed
Description of data collection	Sensor data were collected continuously from February to June 2023. Continuous waveforms were trimmed around the origin time of earthquake events, recorded around the city of Pollenza (MC), Italy, near to the installation site. The events were selected with the following criteria: • Events of any magnitude in a radius of 10 km around Pollenza; • Events of magnitude higher than 2.5 in radius of 50 kms around Pollenza. and then further subsampled depending on data availability.
Data source location	Wireless Sensor Network installed on a building in the Marche Region (Italy), located at the coordinates: latitude 43.24957411513644, longitude 13.395272274610832. The event-related information included in the metadata was obtained from the INGV Earthquakes catalog at http://terremoti.ingv.it/
Data accessibility	Repository name: Mendeley Data Data identification number: 10.17632/zsrk4cngtr.2 Direct URL to data: https://data.mendeley.com/datasets/zsrk4cngtr/2
Related research article	The preliminary validation of the MEMS unit used in this experiment was carried out in this related paper: P. Pierleoni, A. Belli, M. Esposito, R. Concetti and L. Palma, “Earthquake Early Warning Services Based on Very Low-Cost Internet of Things Devices,” 2022 61st FITCE International Congress Future Telecommunications: Infrastructure and Sustainability (FITCE), Rome, Italy, 2022, pp. 1-5, doi: 10.23919/FITCE56290.2022.9934792. https://ieeexplore.ieee.org/document/9934792/

## 1 Value of the Data

• The dataset enables the development of machine learning and deep learning models and algorithms for automating seismic processing tasks, including phase picking, classification, and detection of earthquakes.
• This dataset stands apart from conventional earthquake waveform datasets that include data from networks or databases managed by national monitoring agencies with higher-quality instrumentation, such as [1,2].
• While other works have evaluated MEMS devices for structural monitoring [3], seismology [4], and analysis of Italian seismicity [5], [6], [7], [8], this dataset has been tailored for the development of ML applications. It can be utilized to develop models and algorithms based on carefully annotated, low-cost sensor data acquired during seismic events.
• The dataset and data collection experiment could provide insights into the application of cost-effective technologies in the fields of seismology, geoscience, and civil engineering.
• The dataset also includes a small subset of noise-only records, mainly consisting of the sensors’ own electronic noise, environmental, and anthropic noises. Those might also be useful for research on detection algorithms.

## 2 Objective

The dataset was created to provide researchers with an annotated collection of earthquake waveforms acquired from low-cost MEMS sensors. It has the objective of aiding the development of algorithms based on acceleration signals with low signal-to-noise ratios. Machine Learning (ML) techniques have been increasingly applied to seismology and civil engineering applications, for example to automate the picking of P and/or S waves from waveform data. ML models for these tasks are usually trained on public datasets [1,2]. Such datasets contain traces from strong motion and/or weak motion instrumentation belonging to networks managed by national agencies, consisting mostly of high-quality instrumentation. MEMS and Wireless Sensor Networks (WSNs) have been used in fields such as Structural Health Monitoring and Early Warning Systems [3]. They have demonstrated potential for seismology applications [4] and for integration into existing seismic [5,6] and urban monitoring networks [7]. They have been directly tested on Italian seismicity in multiple studies [5], [6], [7], [8].

This dataset includes recordings from very low-cost MEMS devices of low and moderate magnitudes. Noise records are also included. The dataset is designed to aid the development of algorithms exploiting low-cost technologies, and provide insights about their application in seismology and civil engineering.

## 3 Data Description

The data consists of two datasets, one containing earthquake traces, the other containing noise-only traces.

### 3.1 Earthquake data description

The earthquake dataset consists of a dataset_earthquakes folder and a miniSEED_files folder. The first folder contains an HDF5 file named waveforms.hdf5 and a CSV file named metadata.csv. The miniSEED_files folder contains miniSEED files.

The waveforms.hdf5 file holds the seismic traces, while the metadata.csv file contains metadata and additional information associated with each trace. The miniSEED_files folder contains the miniSEED files that were analyzed and annotated before exporting them to HDF5 format.

The traces in the HDF5 file are 3 × 25,001 arrays, each with an associated trace name (‘trace_name’ field in the metadata.csv file). The dataset follows the “Seisbench data format” [9], where each trace in the waveforms.hdf5 file has an associated row in the metadata.csv file. The trace names also follow the Seisbench convention, and are named as *bucket0$trace_number;:n_dimensions;:n_samples*', where bucket0′ indicates the block to which the trace belongs (in this case, there's only one block due to the dataset' relatively small size); 'trace_number' indicates the trace' index within the block; 'n_dimensions' denotes the number of measurement axes; and 'n_samples' represents the number of samples in the trace.

As a miniSEED file might only contain data for one measurement axis, each trace in the HDF5 file corresponds to three files in the miniSEED_files folder. The names of the three miniSEED files that comprise a trace in the HDF5 file are indicated in the metadata row of that trace, under the ‘trace_name_original_1’, ‘trace_name_original_2’, and ‘trace_name_original_Z’ fields in the metadata.csv file. miniSEED file names follow the format '_eventID_originTime_WS.POZA.Sx.DNy.MSEED', where eventID is the ID of the event that is recorded in the trace, originTime is the origin of the event expressed in UTCTime (YYYY-MM-DDThh:mm:ss.ssssss format), x is a number that is used to identify the sensor that recorded the trace, y indicates the measurement direction of that trace, named '1′, '2′, 'Z'. Details on the measurement axes directions will be given in the Experimental Design and Data Assemble Sections.

As mentioned above, each trace in the dataset has rich metadata associated with it. The metadata can be divided into the following categories: source metadata, sensor metadata, and trace metadata.

- Source metadata describes the earthquake event recorded in the trace, including its focal parameters (origin time, hypocenter coordinates, magnitude, magnitude type, and so on), and the eventXML identifier for that event on the INGV FDSN web-service (https://www.eida.ingv.it/).
- Sensor metadata includes the sensor name and the sensor orientation. The sensor orientation is expressed according to the azimuth and dip convention.
- Trace metadata describes characteristics of the trace, such as amplitude maximum and minimum values, root mean square value, and so on. Each metadata row has trace metadata for all three acquisitions along the accelerometer measurement axes, indicated by the suffixes ‘1’, ‘2’ and ‘Z’, respectively. The axes orientation corresponding to each of the suffixes is detailed in the Experimental Design and Data Assembly Sections. The “components_order” field specifies the relationship between the measurement axes and the dimensions of the arrays in the hdf5 file. The “trace_name_original_x” fields (where x indicates the measurement axes suffixes) show the name of the miniSEED file in the miniSEED_files folder which contain the measurement for each measurement axis of that trace. The trace metadata also includes the picks metadata.

The INGV analysts were able to annotate phases with magnitudes until 1.0 in the near field, with varying degrees of confidence. In some events (mostly for low magnitudes and/or high distances from the source), the phases were not distinguishable at all on any trace. These traces were still maintained as they could be useful to researchers. This conservative approach is in line with another dataset of Italian seismic events for ML, the INSTANCE dataset [1]. Noise traces and low-quality acquisitions can indeed be useful for the development of dedicated models with noisy acquisitions, missing data [10], or even for the development of dedicated denoising algorithms [11]. Either way, users can filter out these traces using the metadata fields associated with the pick times for P and S waves (which are left empty in the metadata.csv file when the pick is not available for that trace).

The uncertainty values associated with manual picks (when available) and their meanings are described below. The errors for P and S picking are expressed in seconds. They represent gaussian errors centered around the annotated pick time.

**Fig. 1:**
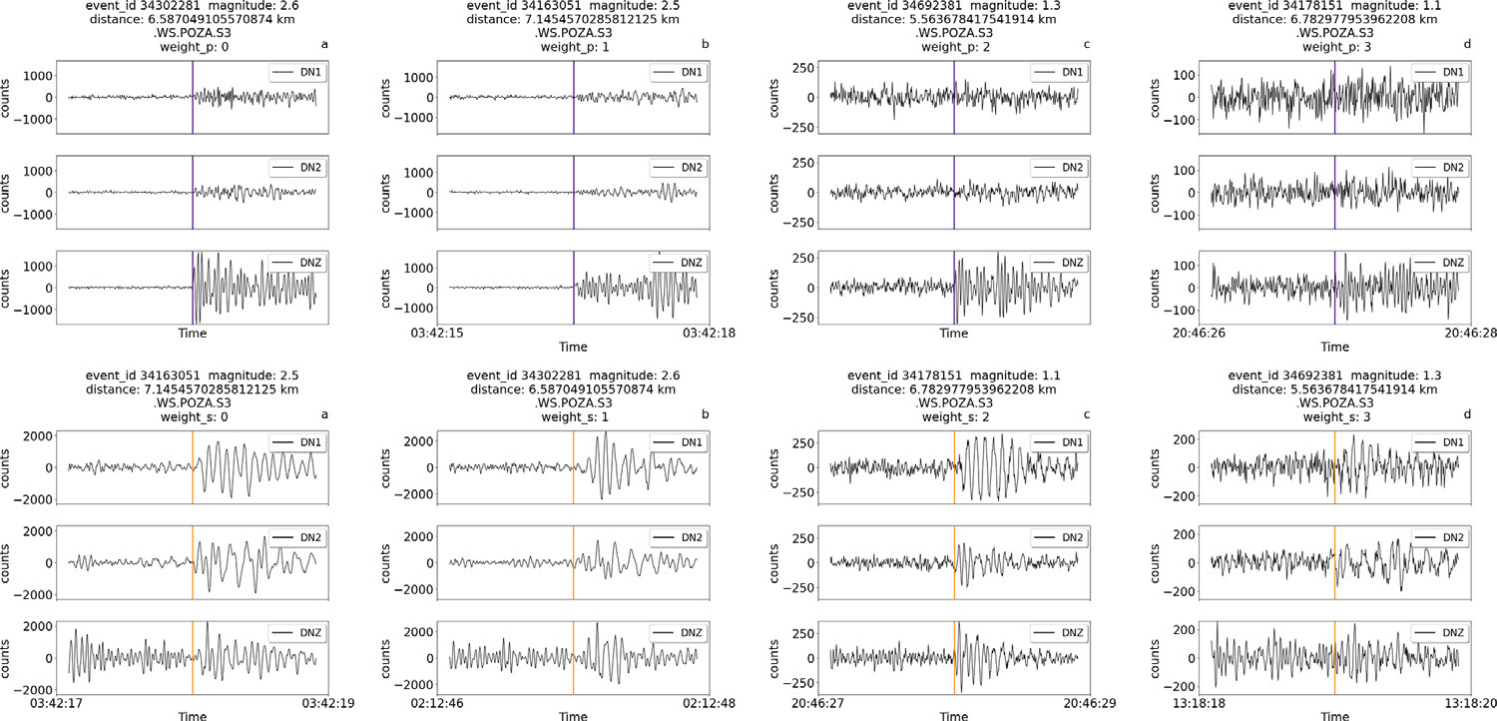
Zoomed-in traces and relative metadata, including the weight associated to each manual annotation. Each trace lasts 2 seconds around the P pick (first row) or the S pick (second row). The picks for P and S onsets are indicated in purple and yellow, respectively. The annotation for the P wave was done on the DNZ channel, the annotation for the S wave on the DN1 and DN2 channels.

Fig. 1 shows examples of traces in the dataset zoomed around the P wave pick, starting one second before the P wave pick and ending three seconds after the P wave pick. The traces were all chosen from the same sensor node. For each trace, the identifier for that event (event_id), magnitude, distance from the event, sensor name, and weights for the P wave pick and S wave pick are also shown (indicated as weight_p and weight_s in the figure, respectively).

**Fig. 2:**
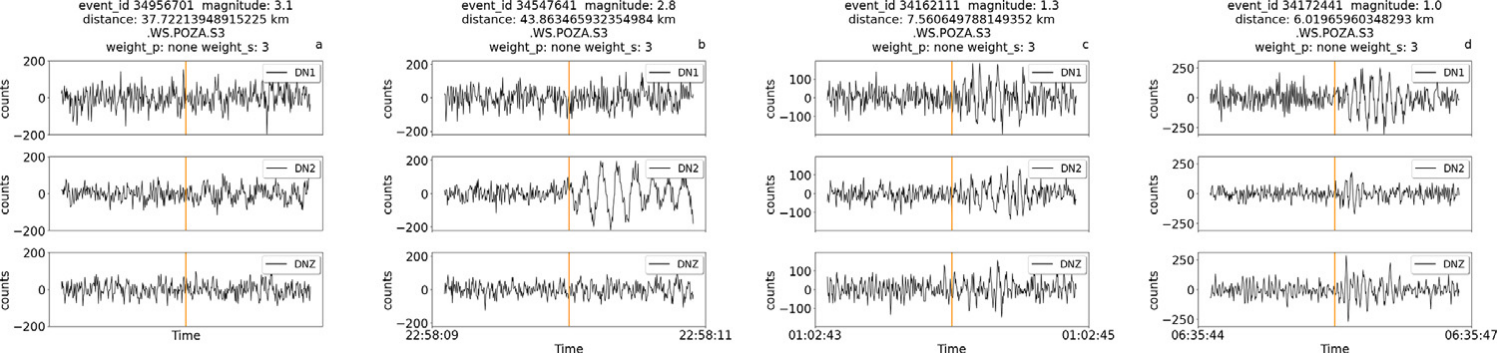
Zoomed-in traces and relative metadata, including the weight associated to each manual annotation. The traces were chosen among low-quality traces with high error weights. Each trace lasts 2 seconds. The picks for S onsets are indicated in yellow.

Fig. 2 shows examples of traces zoomed around the S pick for low quality traces. In this case a manual P pick is not available because of the low signal-to-noise ratio, and therefore no annotation is shown for the P-phase, while the S pick is not interpretable, but it can be guessed from the trace and event data. Most of these traces are relative to either quite distant events (further than 35 km from the installation site) or weak motions (around magnitude 1). In this Figure and in the following the absence of annotations for either the P, S wave, or both, is indicated by assigning the value ‘none’ to the weight field in header title of each trace.

The previous Figures illustrate the different weights that can be associated to picks, highlighting the role of distance and magnitude in determining the uncertainty of annotations. High magnitude events and/or events in the vicinity of the site display lower weights (higher accuracy), while the opposite happens for distant or weak events. As events become weaker and/or further from the site, the onset time of the P wave becomes undistinguishable from the background noise and the traces lack the P pick annotation, as shown in Fig. 2. In this case, the metadata fields for both the uncertainty and the pick timestamp are left empty and can therefore be filtered out with appropriate routines. In other cases, both the P and S waves picks are not available.

**Fig. 3:**
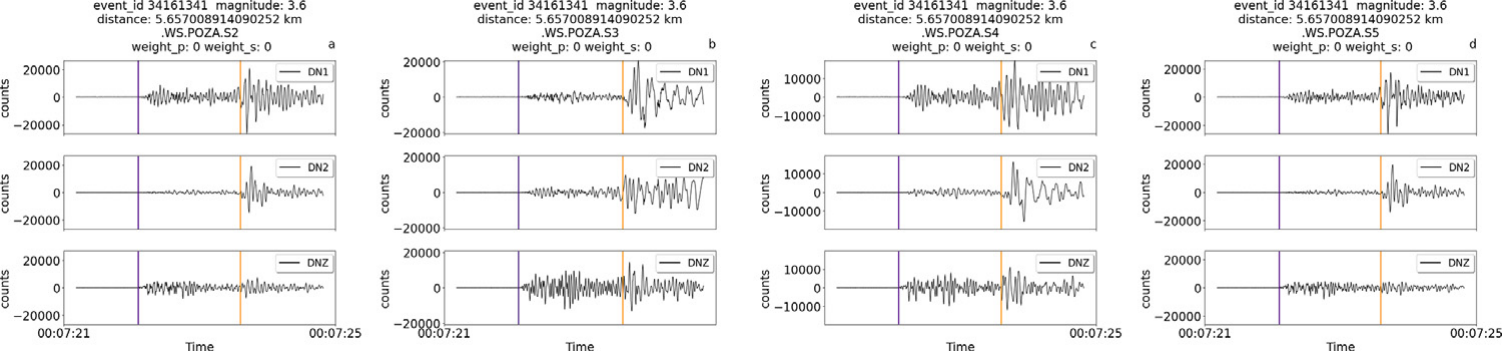
Traces for the event of 3.6 magnitude of February 21st 2023 (event ID 34161341) at 5.65 km from the experiment.
ii. Manual picks for a series of lower magnitude events near the site with annotations with relatively good weights, either for S-wave only or for both P and S waves (Fig. 4).

The following images provide further examples of different types of traces, this time taken from different sensors across the installation site. Each trace is 4 seconds long. In particular, the images show examples of annotations belonging to the following scenarios:

i. Manual picks available for both P and S waves for a relatively strong magnitude event (3.6 magnitude) near the site at 5.65 km (Fig. 3)

**Fig. 4:**
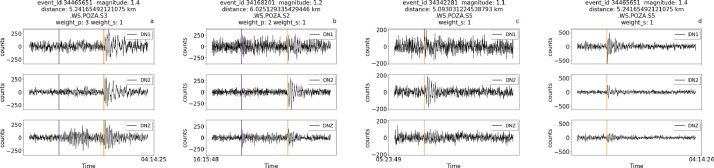
Traces for lower magnitude earthquakes with P and S manual picks
iii. No available manual picks belonging to far and/or low magnitude events (Fig. 5).

**Fig. 5:**
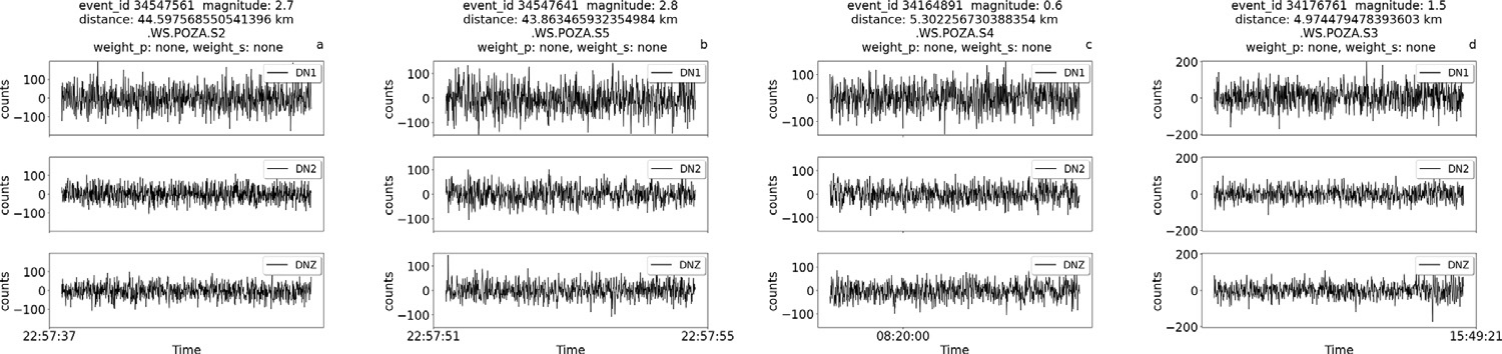
Traces with no available manual picks. The traces have been trimmed around the origin time of the event.
iv. Manual picks available for both S and P waves for a relatively strong magnitude event (3.3 magnitude) that is also relatively far from the site, at about 33 km (Fig. 6). In this case, due to the distance, only the pick for the S wave is available, with higher uncertainty compared to the event in Fig. 3. The uncertainty level in the header of the figure (weight=2) indicates that the picks are visible in the trace but are difficult to interpret.

**Fig. 6:**
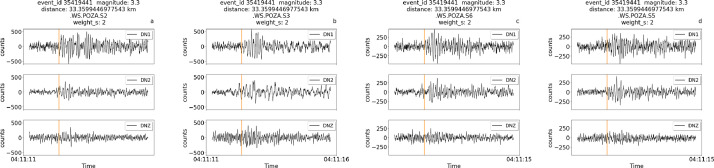
Traces for the event of 3.3 magnitude of June 30th 2023 (event ID 35419441) at relatively far distance(33 km) from the experiment.

Each figure also displays the associated weight for P picks and/or S picks, when available.

**Fig. 7:**
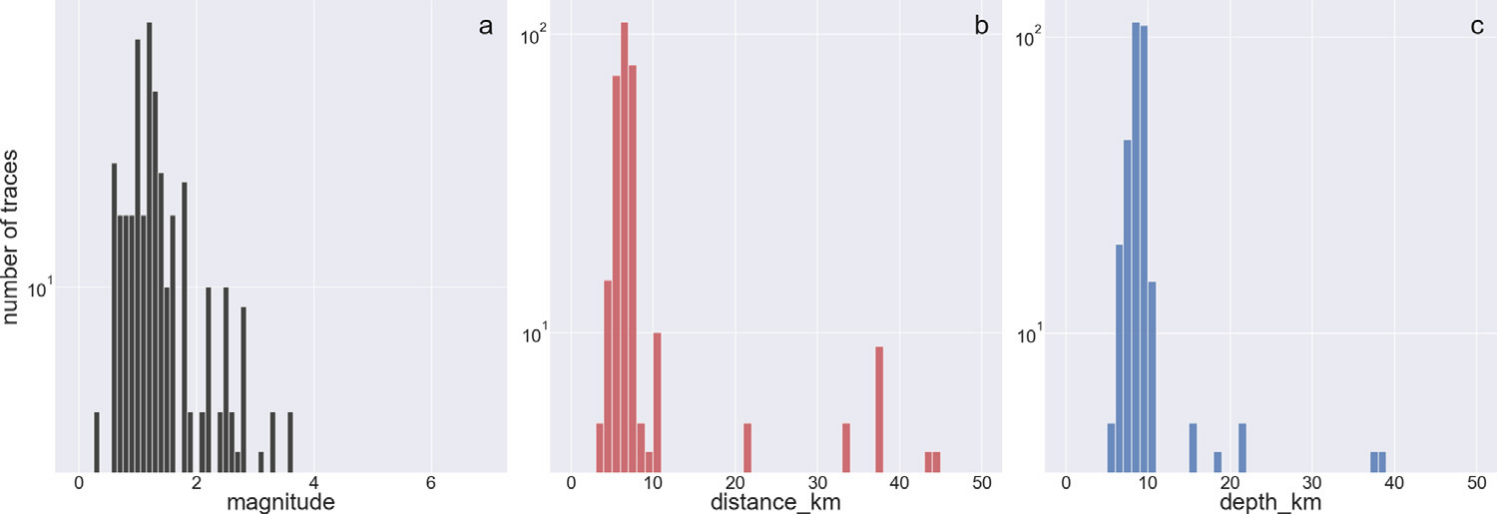
Distributions of the earthquake traces in the dataset according to magnitude, sensor distance from the epicenter, and event depth.

**Fig. 8:**
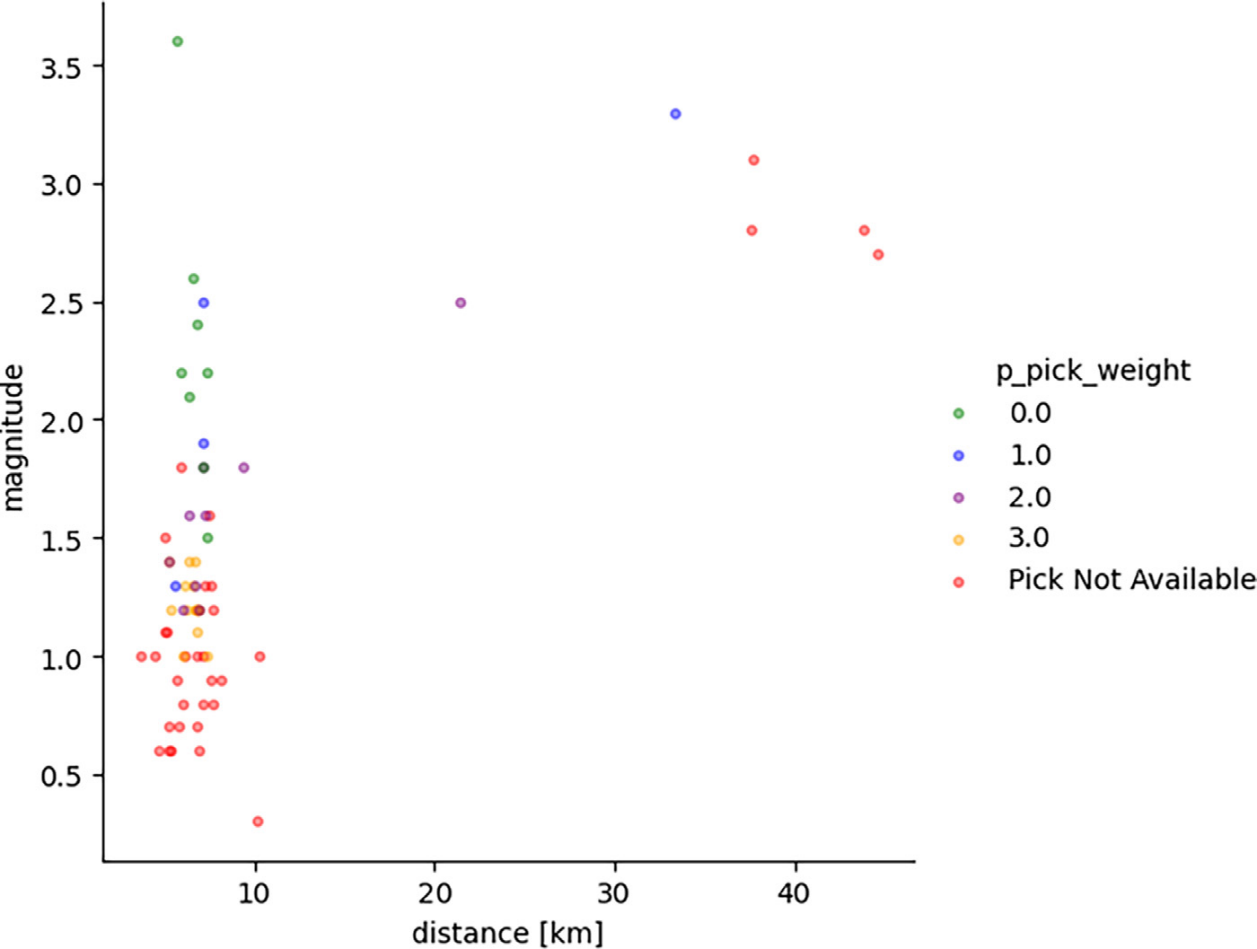
Uncertainty for P picks as a function of event magnitude and distance.

**Fig. 9:**
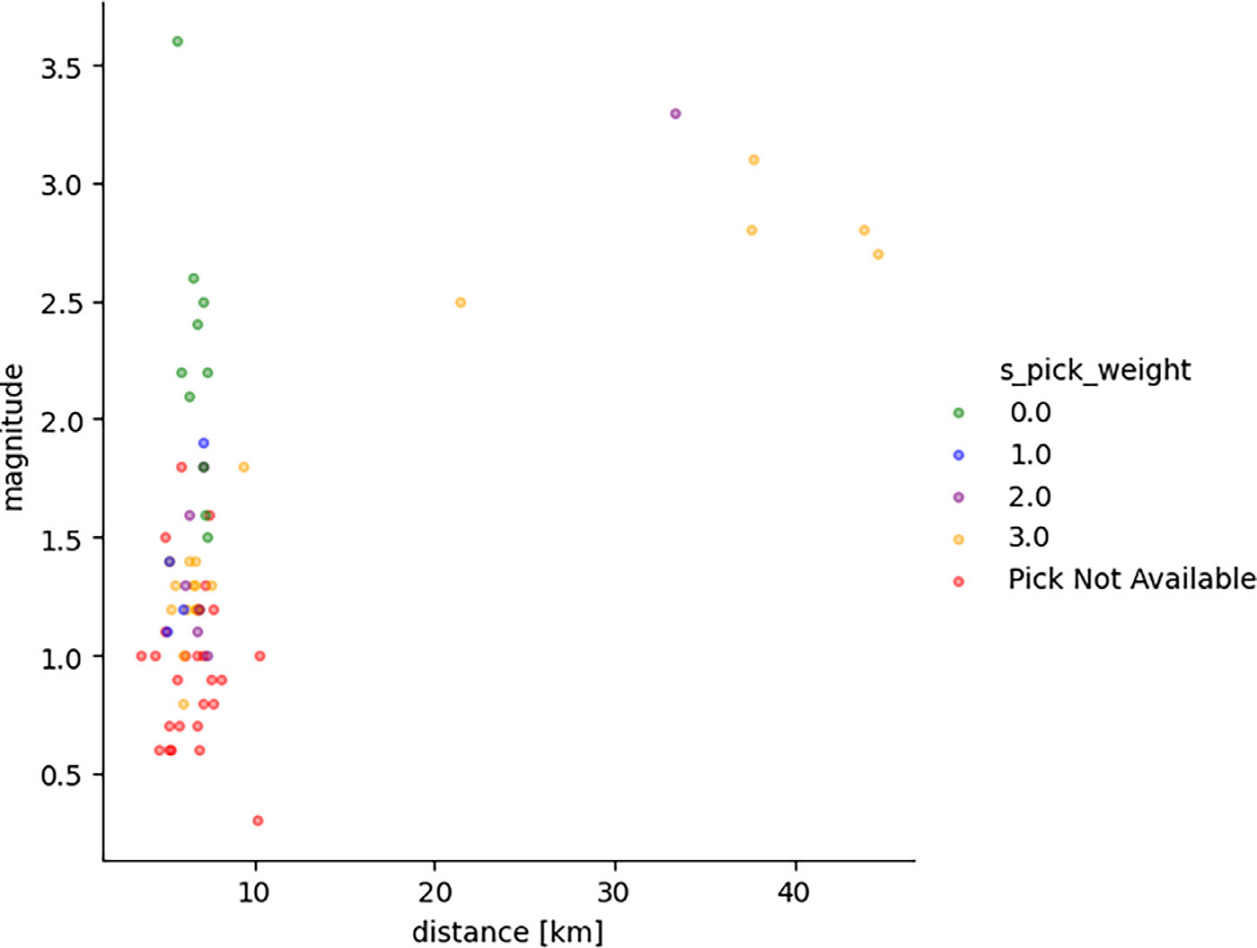
Uncertainty for S picks as a function of event magnitude and distance.

**Fig. 10:**
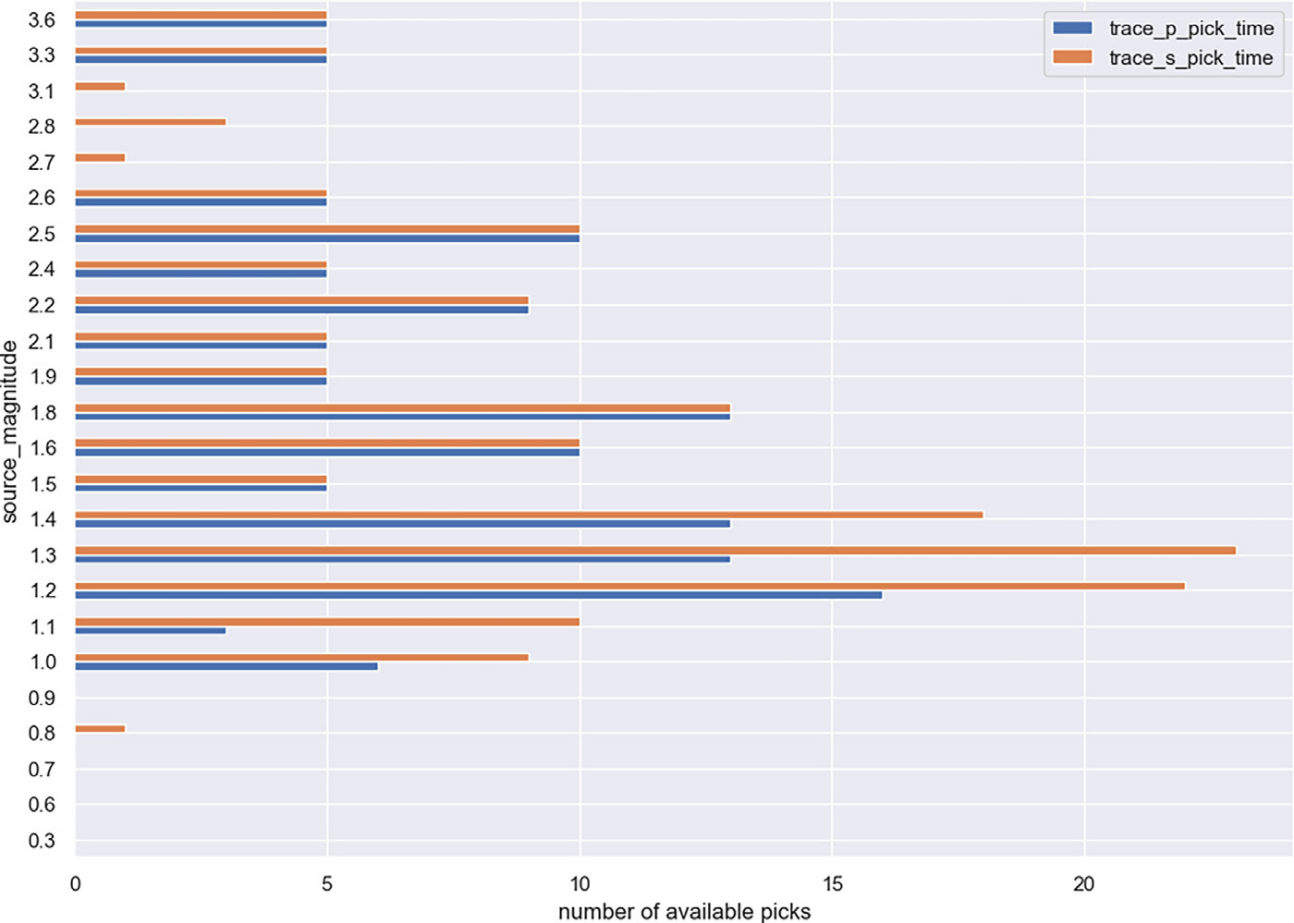
Phase pick availability for the P and S waves for each recorded magnitude.

**Fig. 11:**
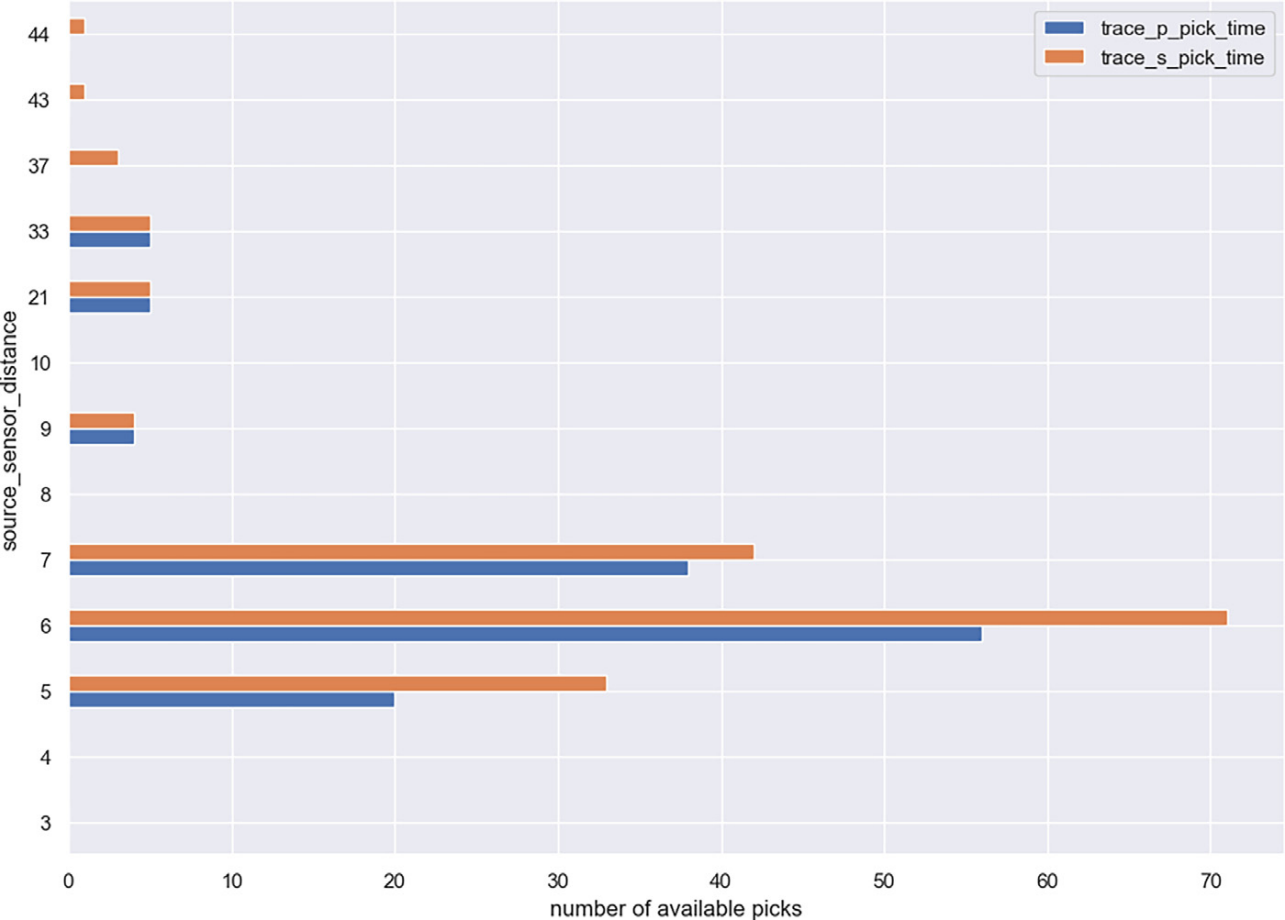
Picks annotations availability for the S and P waves for each source/sensor distance value in the dataset. The distance is expressed in kms.

The following figures provide some general statistics on the dataset. Fig. 7 displays the distribution of the earthquake records in the dataset according to the events magnitude, depth, and the distance between the events and the locations of the wireless sensor network. The minimum magnitude in the dataset is 0.3, while the maximum magnitude is 3.6. The minimum distance from the earthquake source is 3.6 km, while the maximum distance is 44.59 km. Fig. 8 illustrates the uncertainty for P and S picks as a function of event magnitude, while Fig. 9 depicts the uncertainty for P and S picks as a function of event distance from the installation site. Fig. 10, Fig. 11 show the number of available manual picks for each magnitude and distance in the dataset for S and P picks, respectively. To better showcase pick availability in relation to distance and magnitude, when there is more than one pick at the same magnitude/distance coordinate, only the highest weight is displayed.

Figures from 7 to 11 highlight the relationship between distance and accuracy of the annotation, as higher distances always correspond to worse weights or total absence of a manual annotation. Similar considerations hold true for magnitudes. The minimum magnitude with a P pick annotation is 1, even if it has the highest uncertainty, while lower magnitudes have no available annotations. Moving to higher magnitudes, phases start become more easily distinguishable and lower weights are associated to annotations for both P and S waves. Similarly, at distances above 40 km, only some S-pick annotations are available, but with a high weight (meaning the phase is not interpretable but the analyst was able to guess it from the trace and event information).

### 3.2 Noise data description

The noise dataset consists of a folder named “dataset_noise” and a folder named miniSEED_files_noise. The first folder contains an HDF5 file named waveforms.hdf5, and a CSV file named metadata.csv. The second folder contains miniSEED files that were used to create the HDF5 dataset.

The noise data follows the same conventions as the other dataset. As these traces are not relative to any seismic event, the metadata does not contain event or pick information. Trace statistics are included. There is a total of 43 noise traces, each 25001 samples long. Those contain contributes of electronic noise, and environmental and anthropic noises that are picked up by the sensors. To show the effect of anthropic noises, both diurnal and nocturnal acquisitions are included.

Examples of noise traces are reported in the following figure.

## 4 Experimental Design, Materials and Methods

### 4.1 Experimental design

The WSN used to collect data in the experiment was installed in a site in the Marche Region, precisely near the city of Pollenza, Italy. The site is a newly built building made of press-folded steel. The WSN has been installed on the site to monitor its structural health and also to verify the suitability of the low-cost sensors to record nearby earthquake sources. Fig. 13 shows the position of the installation site on a map, as well as the relative position of the two closest cities (Pollenza and Macerata) and the location of the 67 events recorded and included in this dataset.

The network that has been installed is comprised of 5 wireless nodes and a sink node. The wireless nodes embed an ADXL355 accelerometer [12] and continuously transmit accelerometer data towards the sink node through the MQTT protocol [13,14]. To protect and house the nodes, custom utility access boxes were constructed within the building's walls to place the sensors, ensuring direct contact with the steel structure. Fig. 14 shows the sensors positions on the building and the orientation of the sensors’ axes. Fig. 15 depicts the sensor node with and without the external plastic box, as well as the placement of a sensor inside a wall.

A sink node is used to collect data from all five sensors and convert it to miniSEED format. It consists of an embedded PC that runs an MQTT broker, a dedicate proxy for miniSEED conversion, and a ringserver [15] to make the data available to remote clients through the Seedlink protocol. A remote Seedlink client based on the SeiscomP [16] software has been used to acquire and store data in a daily format.

### 4.2 Data assembly

Data were continuously acquired from February 2023 to June 2023 in miniSEED format, with a fixed sampling frequency of 250 Hz.

In order to obtain traces centered around the origin time of earthquake events nearby the installation site, a catalog of events in QuakeQML format was created querying the INGV web service (http://terremoti.ingv.it/).

A first query used the following parameters

○ Start time: 21st February 2023
○ End time: 23rd May 2023
○ Region type: circular
○ Region radius: 10 km
○ Region centre: 43,2688 latitude, 13,3485 longitude

and provided a total catalog of 77 events with magnitudes ranging from 0.3 to 3.6. This query provided events in an “on-site” monitoring range. However, due to issues in the continuous data acquisition system, not all events/days in the query were available in our centralized server, or they included missing data points. As a result, these events were excluded during the subsequent analysis and annotation as they were unavailable.

A second query was conducted to include events further away from the site, to verify the capability of the proposed solution for the detection of events that are further from the site but still relatively strong in terms of magnitude. As such, a minimum magnitude threshold was set for this query and the radius value was raised to 50 km. This second query used the following parameters.

○ Start time: 21st February 2023
○ End time: 30th June 2023
○ Region type: circular
○ Region radius: 50 km
○ Region centre: 43,2688 latitude, 13,3485 longitude
○ Minimum Magnitude: 2.5

This query bore a total of 10 results across 8 different days, some of which were already included in the first query. Considering the missing data and the overlapping days between the two queries, the final dataset included a total of 67 events, with magnitudes ranging from 0.3 to 3.6.

Based on this final catalog of events, the continuous data was trimmed using the Obspy Python library [17]. Particularly, 25,001 samples were taken for each event, starting 20 s before the origin time of the event, and ending 80 seconds after the origin time. Since the site is close or relatively close to the events, the chosen window is large enough to include both the P wave and the S wave onsets, besides several seconds of trace after the event.

The trimmed data was then detrended to remove gravitational offset.

The resulting traces were saved in miniSEED format and P/S wave onsets were annotated by human analysts from the INGV. The annotations consisted of an arrival time and an uncertainty level associated to the pick, dependent on the clearness of the phase onset compared to the background noise, as previously described.

The annotated traces were then processed and converted to HDF5 format using the Seisbench Python package (Woollam et al., 2022). Contextually, additional metadata were computed on each trace and event. In particular, the event metadata for each trace were obtained directly from the QuakeQML catalog, while the trace information was obtained using the MSEEDMetadata Obspy class, which provides methods to automatically analyze miniSEED files. The complete data assembly steps are illustrated in Fig. 16.

**Fig. 12:**
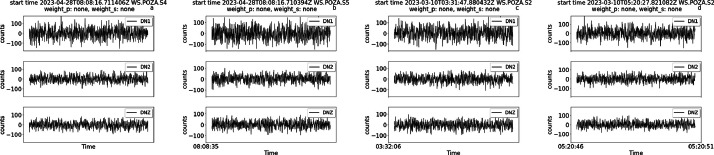
Examples of noise-only traces. The first two traces are relative to day-time acquisitions, the second two to night-time acquisitions.

The noise traces collection followed a similar process. 4 days were chosen among the days in the catalog, and random windows were chosen outside of the seismic events. For each day, both a diurnal and nocturnal window were chosen, as anthropic noise experiences variations throughout the day. After trimming, the traces were processed and converted to h5 and .csv, with no manual annotation as they do not contain seismic events (Fig. 12,Table 1).

**Fig. 13:**
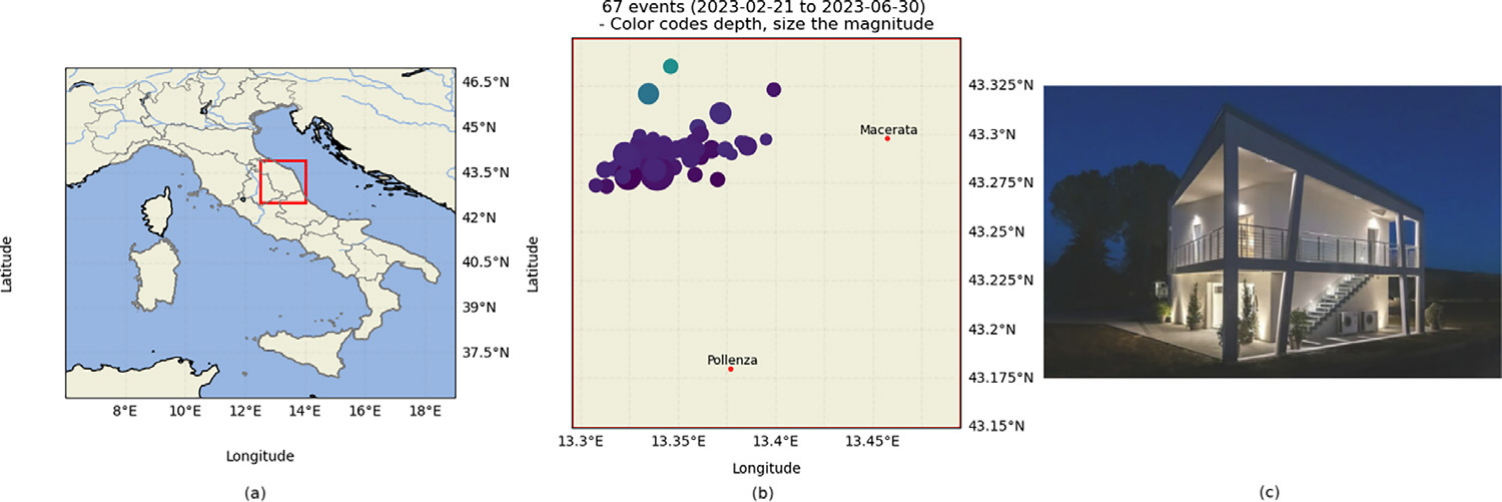
Installation site position, showing the position of the WSN in relation to the cities of Macerata and Pollenza (a), the geographical distribution of 67 events that were included in the dataset (b), and a view of the installation site (c).

**Fig. 14:**
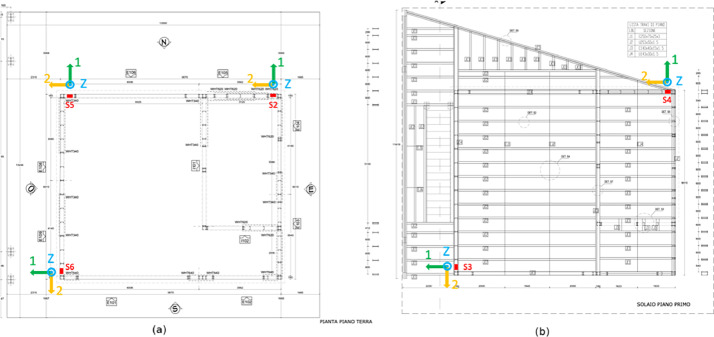
Position of the 5 sensors across the building, depicting the planimetry of the structure and the orientation of each sensor on the ground (a) and first floor (b). The name of the axes refers to the name and orientation of the traces as outlined in the final dataset, which are detailed in the Data Assembly Section.

**Fig. 15:**
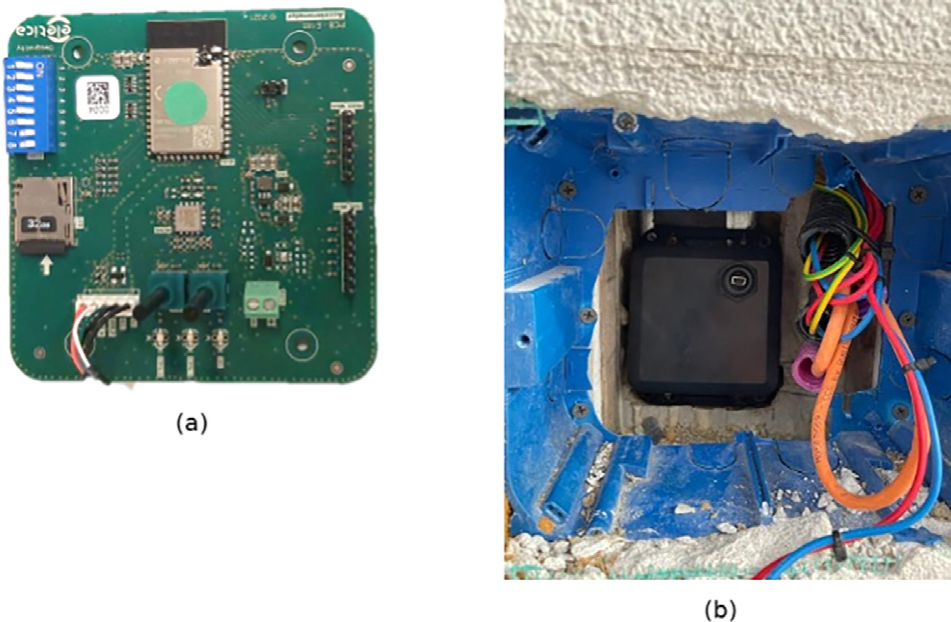
Top view of the sensor node containing the ADXL355 accelerometer used in the experiment (a); a view of a sensor node being installed in its utility box for the experiment (b).

**Fig. 16:**
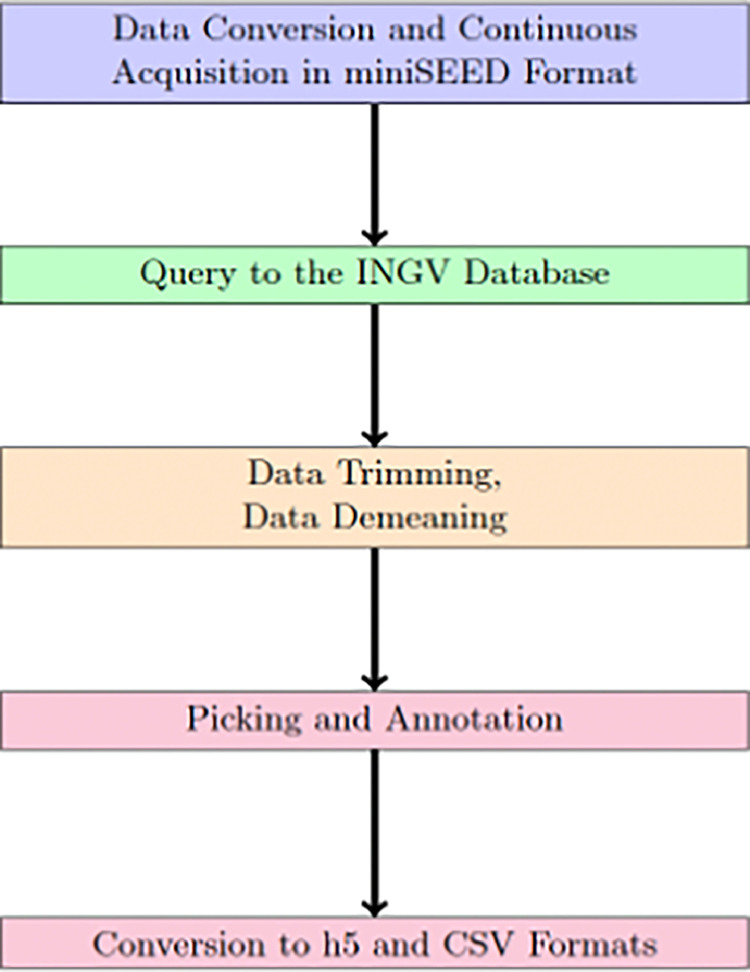
Flow of the data collection and assembly procedure.

**Table 1:** Uncertainty levels for picks annotations and their explanation.

Weight	Error P(s)	Error S(s)	Quality
0	0.0300	0.0600	The phase can be easily distinguished from background noise, and it can be interpreted with high accuracy.
1	0.0600	0.1200	The phase can be interpreted, but the arrival time is uncertain
2	0.2500	0.5000	The phase can be seen in the trace, better if applying a filter, but it is difficult to interpret
3	0.5000	1.5000	The phase is not interpretable, but it can be guessed from the trace and event information

## Limitations

The dataset in its current form has limitations regarding its size due to the limited number of seismic events that occurred near the installation site. Similar experiments will be carried out to increase its size in the future.

## CRediT authorship contribution statement

**Marco Esposito:** Conceptualization, Software, Validation. **Simone Marzorati:** Conceptualization, Software, Formal analysis, Data curation. **Alberto Belli:** Conceptualization, Software, Formal analysis. **Chiara Ladina:** Formal analysis. **Lorenzo Palma:** Conceptualization, Software. **Carlo Calamita:** Investigation. **Debora Pantaleo:** . **Paola Pierleoni:** Methodology, Software, Investigation, Visualization.

## Data Availability

Dataset of signals acquired from MEMS accelerometers during seismic events in Central Italy (Original data) (Mendeley Data)
